# Supplementary material for: Excitatory drive to the globus pallidus external segment facilitates action initiation in non-human primates
Source: bioRxiv. 2025 Apr 21:2025.04.16.649214. Preprint. [Version 1] doi: 10.1101/2025.04.16.649214 (PMC12191113; doi:10.1101/2025.04.16.649214)
Supplement: Supplement 1 [file media-1.pdf]

Supplementary Methods
Detailed model specifications
Model specifications for the statistical analyses presented in the main text are detailed below. All linear mixed-effects (LMM) and generalized linear mixed-effects (GLMM) models included random effects (e.g., [1|monkey_ID] or [1|monkey_ID: Neuron_ID]) to statistically control for individual differences at both the subject and neuron levels. By accounting for these hierarchical structures, the models minimized the risk of inflated type I errors due to the nonindependence of observations within the same monkey or neuron, ensuring more accurate and robust inferences.

1. Linear mixed-effects model for neuronal activity at scene onset (Figures 2B, G, and K)
Model objective: To evaluate differences in neuronal activity following scene onset.

Model specifications:
Full Model: NormalizedNeuronalActivity ~ Scene + (1|monkey_ID) + (1|monkey_ID: Neuron_ID)
Null Model: NormalizedNeuronalActivity ~ (1|monkey_ID) + (1|monkey_ID: Neuron_ID)

Variables:
NormalizedNeuronalActivity: Mean Z-transformed PSTH (100-300 ms after scene onset)
Scene: Fixed effect (levels: 1-4)
monkey_ID, Neuron_ID: Random effects

Statistical threshold: $\alpha = 0.05$

2. Linear mixed-effects model for neuronal activity at target onset (Figures 2D, H, and L)
Model objective: To examine how neuronal activity varies with scene context, object value, and target direction.

Model specifications:
Full Model: NormalizedNeuronalActivity ~ Scene × Value × Direction + (1|monkey_ID) + (1|monkey_ID: Neuron_ID)
Null Model: NormalizedNeuronalActivity ~ (1|monkey_ID) + (1|monkey_ID: Neuron_ID)

Variables:
NormalizedNeuronalActivity: Mean Z-transformed PSTH (100-300 ms after target onset)

Scene: Fixed effect (levels: 1-4)
Value: Fixed effect (levels: good, bad)
Direction: Fixed effect (levels: contralateral, ipsilateral)
monkey_ID, Neuron_ID: Random effects

Multiple comparisons:
6 pairwise comparisons between conditions (good vs. bad, contralateral vs. ipsilateral)
Bonferroni-corrected threshold: $\alpha = 0.05/6$

3. Linear mixed-effects model for neuronal activity at saccade onset (Figures 3B, D, and F)
Model objective: To examine neuronal activity patterns aligned to saccade onset.

Model specifications:
Full Model: NormalizedNeuronalActivity ~ Scene × Value × Direction + (1|monkey_ID) + (1|monkey_ID: Neuron_ID)
Null Model: NormalizedNeuronalActivity ~ (1|monkey_ID) + (1|monkey_ID: Neuron_ID)

Variables:
NormalizedNeuronalActivity: Mean Z-transformed PSTH (from 150 ms before to 50 ms after saccade onset)
Scene: Fixed effect (levels: 1-4)
Value: Fixed effect (levels: good, bad)
Direction: Fixed effect (levels: contralateral, ipsilateral)
monkey_ID, Neuron_ID: Random effects

Multiple comparisons:
6 pairwise comparisons between conditions (good vs. bad, contralateral vs. ipsilateral)
Bonferroni-corrected threshold: $\alpha = 0.05/6$

4. Linear mixed-effects model for neuronal activity during choice rejection and fixation (Figures 4E, H, K, N, Q, and T)
Model objective: To compare neuronal activity patterns between different rejection strategies and across choice and fixation tasks.

Model specifications:
Full Model: NormalizedNeuronalActivity ~ Condition × Direction + (1|monkey_ID) + (1|monkey_ID: Neuron_ID)
Null Model: NormalizedNeuronalActivity ~ (1|monkey_ID) + (1|monkey_ID: Neuron_ID)

Variables:
NormalizedNeuronalActivity: Mean Z-transformed PSTH (100-300 ms post-target)
Condition: Fixed effect (levels: return (choice task), stay (choice task), good (fixation task), bad (fixation task))
Direction: Fixed effect (levels: contralateral, ipsilateral)

Multiple comparisons:
6 pairwise comparisons between conditions (good vs. bad, contralateral vs. ipsilateral)
Bonferroni-corrected threshold: $\alpha = 0.05/6$

5. Generalized linear mixed-effects model for saccade reaction times after injection (Figure 5A)
Model objective: To investigate the effects of glutamatergic antagonist injection on saccadic reaction times.

Model specifications:
Full Model: MedianSaccadeReactionTimes ~ Injection × PrePost × Value × Direction + (1|monkey_ID) + (1|monkey_ID: Session_ID)
Null Model: MedianSaccadeReactionTimes ~ (1|monkey_ID) + (1|monkey_ID: Session_ID)

Variables:
MedianSaccadeReactionTimes: Median reaction time per condition
Injection: Fixed effect (levels: antagonist, saline)
PrePost: Fixed effect (levels: pre-injection, post-injection)
Value: Fixed effect (levels: good, bad)
Direction: Fixed effect (levels: contralateral, ipsilateral)
Session_ID: Random effect identifying individual injection sessions

Distribution: Poisson
Rationale for distribution choice: Reaction times are non-negative count data characterized by a right-skewed distribution.

Multiple comparisons:
8 pairwise comparisons (pre vs. post for each condition)
Bonferroni-corrected threshold: $\alpha = 0.05/8$

6. Generalized linear mixed-effects model for chosen action rate after injection (Figure 5B)
Model objective: To investigate the impact of glutamatergic antagonist injection on action selection for bad objects.

Model specifications:
Full Model: ChosenActionRate ~ Injection × PrePost × Value × Direction + (1|monkey_ID) + (1|monkey_ID: Session_ID), weights = (total trial count)
Null Model: ChosenActionRate ~ (1|monkey_ID) + (1|monkey_ID: Session_ID), weights = (total trial count)

Variables:
ChosenActionRate: Proportion of selected actions
Injection, Pre- Post-, Value, Direction: Fixed effects as described above
total_trial_count: Weights to account for different numbers of trials

Distribution: Binomial
Rationale for distribution choice: Analysis of proportional data with binary outcomes

Multiple comparisons:
8 pairwise comparisons (pre vs. post for each condition)
Bonferroni-corrected threshold: $\alpha = 0.05/8$

7. Generalized linear mixed-effects model for fixation break error rate after injection (Figure 5C)
Model objective: To examine how glutamatergic antagonist injection affects the ability to suppress reflexive saccades.

Model specifications:
Full Model: FixBreakErrorRate ~ Injection × PrePost × Value × Direction + (1|monkey_ID) + (1|monkey_ID: Session_ID), weights = (total trial count)
Null Model: FixBreakErrorRate ~ (1|monkey_ID) + (1|monkey_ID: Session_ID), weights = (total trial count)

Variables:
FixBreakErrorRate: Proportion of fixation break errors
All other variables, as defined above

Distribution: Binomial
Rationale for distribution choice: Analysis of error rate data with binary outcomes.

Multiple comparisons:
8 pairwise comparisons (pre vs. post for each condition)
Bonferroni-corrected threshold: $\alpha = 0.05/8$

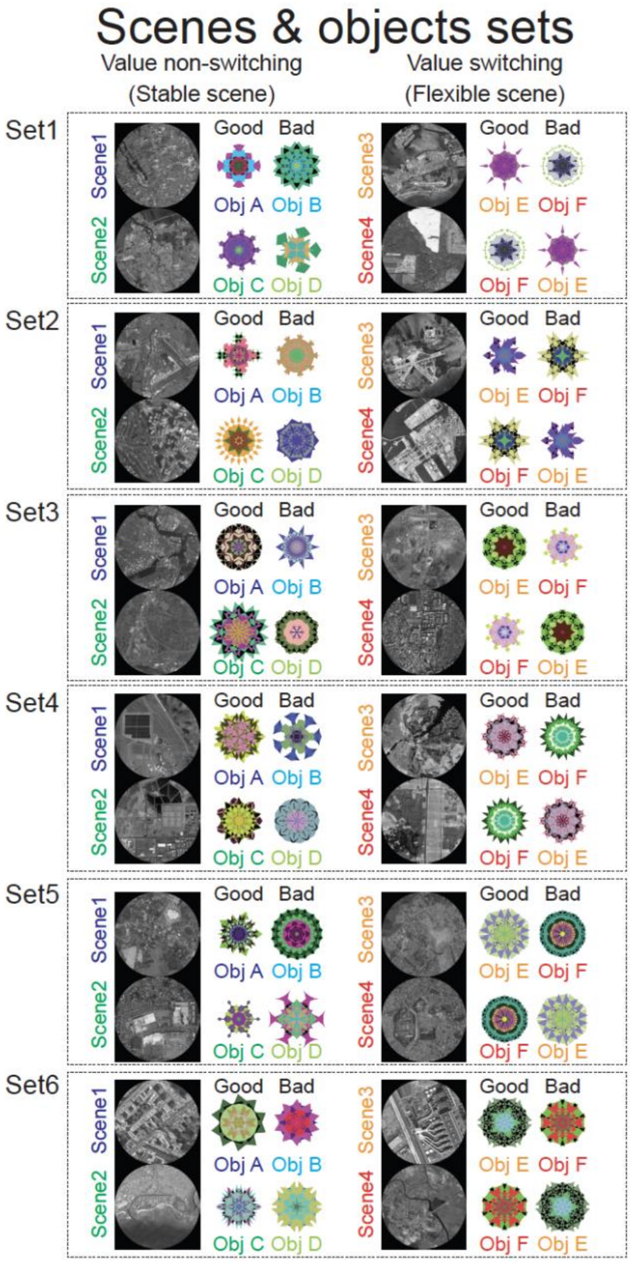

Figure S1. All sets of scenes 1-4 and good and bad objects for the choice task.

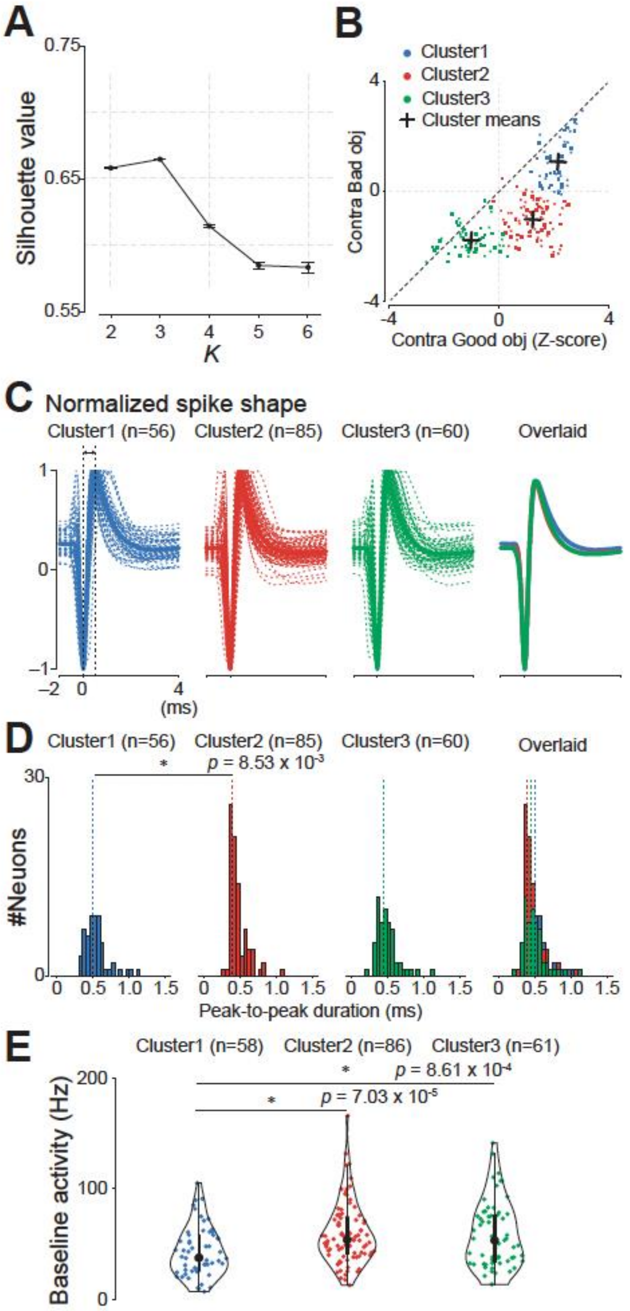

Figure S2. Cluster analysis of GPe neurons based on task-related activity and comparison of their electrophysiological properties.

(A) Determination of the optimal number of clusters (K) for k-means clustering. The graph shows the mean ± SD silhouette values for different K values, calculated from 5,000 simulations. The silhouette value was significantly higher for K=3 than for other values ($p < 0.001$, one-way ANOVA), indicating that the neurons were best classified into three clusters.

(B) Scatter plot of the average standardized firing rates of each neuron during the presentation of a contralateral good object (x-axis) and a contralateral bad object (y-axis). The firing rates were calculated for the period between 100 and 300 ms after object presentation and standardized using the z-score method (see Methods for details). Each dot represents a neuron, and the colors indicate the cluster to which the neuron was assigned: Cluster 1 (blue), Cluster 2 (red), and Cluster 3 (green). The crosses indicate the mean values for each cluster.

(C) Normalized spike shapes for each cluster. The thick lines represent the average spike shape for each cluster, and the thin lines represent the individual spike shapes. The spike shapes were normalized to the trough and peak amplitudes for each neuron.

(D) Histograms of the peak-to-peak duration for each cluster. The peak-to-peak duration was defined as the time between the trough and the peak of the spike waveform. The asterisk indicates a significant difference between Clusters 1 and 2 ($p = 8.53 \times 10^{-3}$, Kruskal–Wallis test followed by Dunn's post-hoc test).

(E) Violin plots of the baseline firing rates for each cluster. The baseline firing rate was defined as the average firing rate during the 500 ms period preceding the scene onset. The format of the violin plots is the same as that in Figure 2B. The larger circle indicates the median value, the thick vertical line shows the interquartile range (IQR), and the thin vertical line indicates the range from the lower to the upper adjacent values (1.5xIQR below the first quartile and 1.5xIQR above the third quartile, respectively). The asterisks indicate significant differences between Clusters 1 and 2 ($p = 7.03 \times 10^{-5}$) and between Clusters 1 and 3 ($p = 8.61 \times 10^{-4}$) (Kruskal–Wallis test followed by Dunn's post-hoc test).

Abbreviations: GPe, external segment of the globus pallidus; SD, standard deviation.

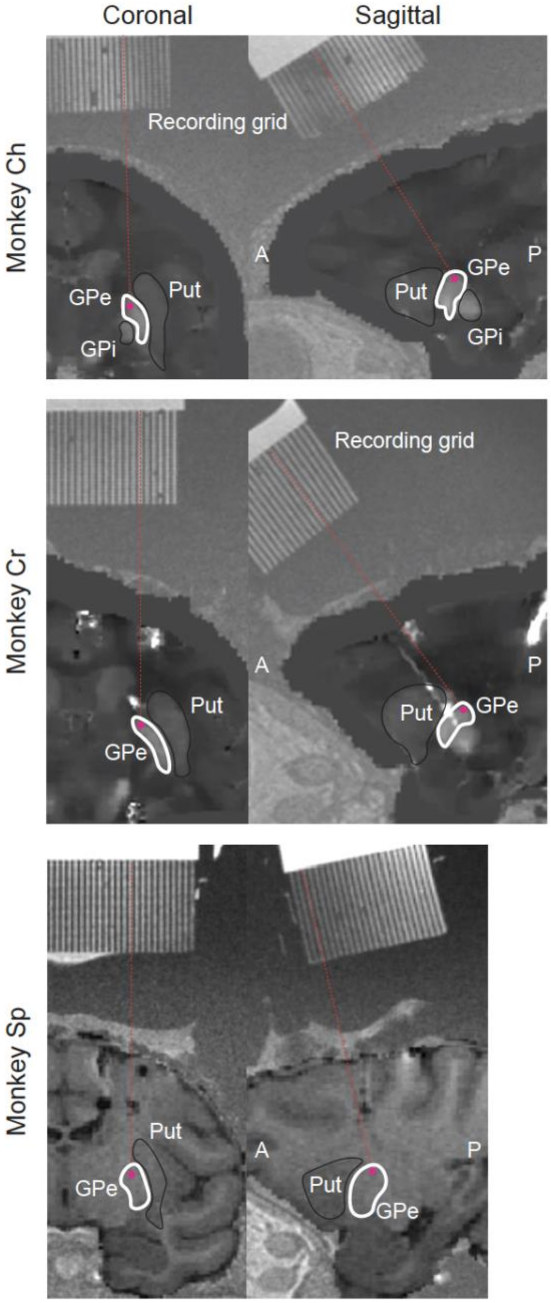

Figure S3. Examples of injection sites in the GPe of three macaque monkeys (Monkey Ch, Monkey Cr, and Monkey Sp).
Coronal (left) and sagittal (right) sections of MRI images show representative injection sites (red dots) in each monkey. The MRI images were fused images of

high-resolution T1-weighted images and either QSM or a conventional three-dimensional T1-weighted image. The white lines delineate the approximate borders of the GPe and putamen. The red dots indicate the location of the injection site, which was targeted to be in the cdGPe. Abbreviations: A, anterior; P, posterior; cdGPe, caudodorsal globus pallidus external segment; GPe, globus pallidus external segment; GPi, globus pallidus internal segment; Put, putamen; QSM, quantitative susceptibility mapping; MRI, magnetic resonance imaging.

Table S1. Saccade reaction times in each condition of each monkey.

**Monkey Cr**	n	mean RT (ms)	SD	95% CI
**Good obj**				
ObjA in Scene1	2176	170.5	23.7	[169.5 171.5]
ObjC in Scene2	2225	171.6	24.2	[170.5 172.6]
ObjE in Scene3	2188	180.0	24.4	[179.0 181.0]
ObjF in Scene4	2163	174.4	22.6	[173.4 175.3]
**Bad obj**				
ObjB in Scene1	1699	250.0	50.4	[247.6 252.4]
ObjD in Scene2	1769	254.1	48.9	[251.8 256.4]
ObjF in Scene3	2003	232.3	51.6	[230.1 234.6]
ObjE in Scene4	1958	235.7	55.2	[233.2 238.1]
**Monkey Sp**	n	mean RT (ms)	SD	95% CI
**Good obj**				
ObjA in Scene1	1960	168.8	24.2	[167.8 169.9]
ObjC in Scene2	1947	174.9	25.9	[173.8 176.2]
ObjE in Scene3	1934	179.0	24.0	[177.9 180.1]
ObjF in Scene4	1932	178.4	25.6	[177.3 179.6]
**Bad obj**				
ObjB in Scene1	1385	284.1	45.5	[281.7 286.5]
ObjD in Scene2	1270	291.4	42.4	[289.0 293.7]
ObjF in Scene3	1629	267.7	54.3	[265.1 270.3]
ObjE in Scene4	1452	280.3	52.1	[277.6 283.0]

Table S2. Counts of chosen actions for Bad objects

**Monkey Cr**	total	accept	Return	Stay	other	fxbreak
Scene1	2219	10	1727	437	3	42
Scene2	2206	4	1791	398	0	13
Scene3	2150	7	2009	119	1	14
Scene4	2255	5	1973	239	0	38
non-switch(scene1,2)	4425	14	3518	835	4	55
switch(scene3,4)	4405	12	3982	358	1	52
**Monkey Sp**	total	accept	Return	Stay	other	fxbreak
Scene1	1927	6	1357	556	0	8
Scene2	1892	9	1246	629	0	8
Scene3	2032	3	1616	407	0	6
Scene4	1816	4	1435	365	0	12
non-switch(scene1,2)	3819	15	2603	1185	0	16
switch(scene3,4)	3848	7	3051	772	0	18

Table S3. Summary of statistical test to compare the normalized neuronal activity of GPe neurons of cluster1 at target onset among conditions during choice task in Figure 2.

Cluster1						
parametric bootstrap test (n = 10,000)	*p*					
full model vs. null model	< .001					
post hoc<br>(pairwise t–test, Bonferroni correction)	Mean (SD)	Mean (SD)	*t*	*p*	95% CI	effect size
**Scene1**						
(good, contra) vs (bad, contra)	2.17(0.44)	1.01(1.17)	8.42	<.0001	[0.90, 1.44]	1.56
(good, contra) vs (good, ipsi)	2.17(0.44)	1.20(0.86)	7.00	<.0001	[0.70, 1.24]	1.30
(good, contra) vs (bad, ipsi)	2.17(0.44)	0.41(1.35)	12.71	<.0001	[1.49, 2.03]	2.36
(bad, contra) vs (good, ipsi)	1.01(1.17)	1.20(0.86)	–1.42	= 1.55 x 10^–1	[–0.47, 0.07]	–0.26
(bad, contra) vs (bad, ipsi)	1.01(1.17)	0.41(1.35)	4.29	<.0001	[0.32, 0.87]	0.80
(good, ipsi) vs (bad, ipsi)	1.20(0.86)	0.41(1.35)	5.71	<.0001	[0.52, 1.06]	1.06
**Scene2**						
(good, contra) vs (bad, contra)	2.23(0.47)	0.95(1.08)	9.23	<.0001	[1.01, 1.55]	1.71
(good, contra) vs (good, ipsi)	2.23(0.47)	1.46(0.75)	5.51	<.0001	[0.49, 1.04]	1.02
(good, contra) vs (bad, ipsi)	2.23(0.47)	0.50(1.07)	12.47	<.0001	[1.46, 2.00]	2.32
(bad, contra) vs (good, ipsi)	0.95(1.08)	1.46(0.75)	–3.73	= 2.00 x 10^–4	[–0.79, –0.24]	–0.69
(bad, contra) vs (bad, ipsi)	0.95(1.08)	0.50(1.07)	3.24	1.20 x 10^–3	[0.18, 0.72]	0.60
(good, ipsi) vs (bad, ipsi)	1.46(0.75)	0.50(1.07)	6.96	<.0001	[0.69, 1.24]	1.29
**Scene3**						
(good, contra) vs (bad, contra)	2.14(0.59)	1.29(1.07)	6.12	<.0001	[0.58, 1.12]	1.14
(good, contra) vs (good, ipsi)	2.14(0.59)	1.24(0.96)	6.50	<.0001	[0.63, 1.17]	1.21
(good, contra) vs (bad, ipsi)	2.14(0.59)	0.58(1.24)	11.24	<.0001	[1.29, 1.83]	2.09
(bad, contra) vs (good, ipsi)	1.29(1.07)	1.24(0.96)	0.38	= 7.05 x 10^–1	[–0.22, 0.32]	0.07
(bad, contra) vs (bad, ipsi)	1.29(1.07)	0.58(1.24)	5.12	<.0001	[0.44, 0.98]	0.95
(good, ipsi) vs (bad, ipsi)	1.24(0.96)	0.58(1.24)	4.74	<.0001	[0.39, 0.93]	0.88
**Scene4**						
(good, contra) vs (bad, contra)	2.15(0.55)	1.17(1.06)	7.05	<.0001	[0.71, 1.25]	1.31
(good, contra) vs (good, ipsi)	2.15(0.55)	1.23(0.98)	6.59	<.0001	[0.64, 1.19]	1.22
(good, contra) vs (bad, ipsi)	2.15(0.55)	0.51(1.08)	11.83	<.0001	[1.37, 1.91]	2.20
(bad, contra) vs (good, ipsi)	1.17(1.06)	1.23(0.98)	0.46	= 6.45 x 10^–1	[–0.34, 0.21]	–0.09
(bad, contra) vs (bad, ipsi)	1.17(1.06)	0.51(1.08)	4.78	<.0001	[0.39, 0.94]	0.89
(good, ipsi) vs (bad, ipsi)	1.23(0.98)	0.51(1.08)	5.24	<.0001	[0.46, 1.00]	0.97

Table S4. Summary of statistical test to compare the normalized neuronal activity of GPe neurons of cluster2 at target onset among conditions during choice task in Figure 2.

Cluster2						
parametric bootstrap test (n = 10,000)	*p*					
full model vs. null model	< .001					
post hoc<br>(pairwise t–test, Bonferroni correction)	Mean (SD)	Mean (SD)	*t*	*p*	95% CI	effect size
**Scene1**						
(good, contra) vs (bad, contra)	1.33(0.68)	–1.11(1.08)	16.50	<.0001	[2.14, 2.72]	2.52
(good, contra) vs (good, ipsi)	1.33(0.68)	0.34(0.94)	6.72	<.0001	[0.70, 1.28]	1.02
(good, contra) vs (bad, ipsi)	1.33(0.68)	–1.13(1.22)	16.67	<.0001	[2.17, 2.74]	2.54
(bad, contra) vs (good, ipsi)	–1.11(1.08)	0.34(0.94)	–9.79	<.0001	[–1.73, –1.15]	–1.49
(bad, contra) vs (bad, ipsi)	–1.11(1.08)	–1.13(1.22)	0.16	= 8.70 x 10^–1	[–0.27, 0.32]	0.02
(good, ipsi) vs (bad, ipsi)	0.34(0.94)	–1.13(1.22)	9.95	<.0001	[1.18, 1.76]	1.52
**Scene2**						
(good, contra) vs (bad, contra)	1.22(0.82)	–1.09(1.01)	15.73	<.0001	[2.03, 2.61]	2.40
(good, contra) vs (good, ipsi)	1.22(0.82)	0.39(1.12)	5.67	<.0001	[0.55, 1.12]	0.86
(good, contra) vs (bad, ipsi)	1.22(0.82)	–1.12(1.05)	15.95	<.0001	[2.06, 2.64]	2.43
(bad, contra) vs (good, ipsi)	–1.09(1.01)	0.39(1.12)	–10.06	<.0001	[–1.77, –1.19]	–1.53
(bad, contra) vs (bad, ipsi)	–1.09(1.01)	–1.12(1.05)	0.22	= 8.29 x 10^–1	[–0.26, 0.32]	0.03
(good, ipsi) vs (bad, ipsi)	0.39(1.12)	–1.12(1.05)	10.28	<.0001	[1.23, 1.80]	1.57
**Scene3**						
(good, contra) vs (bad, contra)	1.31(0.83)	–0.90(1.13)	15.02	<.0001	[1.92, 2.50]	2.29
(good, contra) vs (good, ipsi)	1.31(0.83)	0.52(1.12)	5.43	<.0001	[0.51, 1.09]	0.83
(good, contra) vs (bad, ipsi)	1.31(0.83)	–1.05(1.11)	16.07	<.0001	[2.08, 2.66]	2.45
(bad, contra) vs (good, ipsi)	–0.90(1.13)	0.52(1.12)	–9.59	<.0001	[–1.70, –1.12]	–1.46
(bad, contra) vs (bad, ipsi)	–0.90(1.13)	–1.05(1.11)	1.05	= 2.94 x 10^–1	[–0.13, 0.44]	0.16
(good, ipsi) vs (bad, ipsi)	0.52(1.12)	–1.05(1.11)	10.64	<.0001	[1.28, 1.86]	1.62
**Scene4**						
(good, contra) vs (bad, contra)	1.17(0.88)	–1.05(1.26)	15.04	<.0001	[1.93, 2.50]	2.29
(good, contra) vs (good, ipsi)	1.17(0.88)	0.39(1.10)	5.26	<.0001	[0.49, 1.06]	0.80
(good, contra) vs (bad, ipsi)	1.17(0.88)	–1.19(1.18)	15.98	<.0001	[2.06, 2.64]	2.44
(bad, contra) vs (good, ipsi)	–1.05(1.26)	0.39(1.10)	–9.77	<.0001	[–1.73, –1.15]	–1.49
(bad, contra) vs (bad, ipsi)	–1.05(1.26)	–1.19(1.18)	0.94	= 3.46 x 10^–1	[–0.15, 0.43]	0.14
(good, ipsi) vs (bad, ipsi)	0.39(1.10)	–1.19(1.18)	10.72	<.0001	[1.29, 1.87]	1.63

Table S5. Summary of statistical test to compare the normalized neuronal activity of GPe neurons of cluster3 at target onset among conditions during choice task in Figure 2.

Cluster3						
parametric bootstrap test (n = 10,000)	*p*					
full model vs. null model	< .001					
post hoc (pairwise t–test, Bonferroni correction)	Mean (SD)	Mean (SD)	*t*	*p*	95% CI	effect size
**Scene1**						
(good, contra) vs (bad, contra)	–0.93(1.01)	–1.70(0.86)	6.00	<.0001	[0.52, 1.02]	1.09
(good, contra) vs (good, ipsi)	–0.93(1.01)	–1.14(1.07)	1.59	= 1.13 x 10^–1	[–0.05, 0.46]	0.29
(good, contra) vs (bad, ipsi)	–0.93(1.01)	–1.72(0.83)	6.15	<.0001	[0.54, 1.04]	1.11
(bad, contra) vs (good, ipsi)	–1.70(0.86)	–1.14(1.07)	–4.41	<.0001	[–0.82, –0.32]	–0.80
(bad, contra) vs (bad, ipsi)	–1.70(0.86)	–1.72(0.83)	0.15	= 8.78 x 10^–1	[–0.23, 0.27]	0.03
(good, ipsi) vs (bad, ipsi)	–1.14(1.07)	–1.72(0.83)	4.57	<.0001	[0.33, 0.84]	0.83
**Scene2**						
(good, contra) vs (bad, contra)	–1.14(0.96)	–1.72(0.81)	4.59	<.0001	[0.34, 0.84]	0.83
(good, contra) vs (good, ipsi)	–1.14(0.96)	–1.30(1.02)	1.27	= 2.05 x 10^–1	[–0.09, 0.42]	0.23
(good, contra) vs (bad, ipsi)	–1.14(0.96)	–1.87(0.83)	5.61	<.0001	[0.47, 0.98]	1.02
(bad, contra) vs (good, ipsi)	–1.72(0.81)	–1.30(1.02)	–3.32	= 9.00 x 10^–4	[–0.68, –0.17]	–0.60
(bad, contra) vs (bad, ipsi)	–1.72(0.81)	–1.87(0.83)	1.05	= 2.96 x 10^–1	[–0.12, 0.39]	0.19
(good, ipsi) vs (bad, ipsi)	–1.30(1.02)	–1.87(0.83)	4.35	<.0001	[0.31, 0.81]	0.79
**Scene3**						
(good, contra) vs (bad, contra)	–1.06(0.89)	–1.78(0.87)	5.60	<.0001	[0.47, 0.97]	1.01
(good, contra) vs (good, ipsi)	–1.06(0.89)	–1.33(0.99)	2.08	= 3.82 x 10^–2	[0.01, 0.52]	0.38
(good, contra) vs (bad, ipsi)	–1.06(0.89)	–1.76(0.90)	5.45	<.0001	[0.45, 0.95]	0.99
(bad, contra) vs (good, ipsi)	–1.78(0.87)	–1.33(0.99)	–3.52	= 5.00 x 10^–4	[–0.70, –0.20]	–0.64
(bad, contra) vs (bad, ipsi)	–1.78(0.87)	–1.76(0.90)	–0.15	= 8.85 x 10^–1	[–0.27, 0.23]	–0.03
(good, ipsi) vs (bad, ipsi)	–1.33(0.99)	–1.76(0.90)	3.38	= 8.00 x 10^–4	[0.18, 0.69]	0.61
**Scene4**						
(good, contra) vs (bad, contra)	–1.00(1.01)	–1.83(0.94)	6.47	<.0001	[0.58, 1.08]	1.17
(good, contra) vs (good, ipsi)	–1.00(1.01)	–1.20(1.01)	1.55	= 1.21 x 10^–1	[–0.05, 0.45]	0.28
(good, contra) vs (bad, ipsi)	–1.00(1.01)	–1.78(0.94)	6.07	<.0001	[0.53, 1.03]	1.10
(bad, contra) vs (good, ipsi)	–1.83(0.94)	–1.20(1.01)	–4.91	<.0001	[–0.88, –0.38]	–0.89
(bad, contra) vs (bad, ipsi)	–1.83(0.94)	–1.78(0.94)	–0.39	= 6.95 x 10^–1	[–0.30, 0.20]	–0.07
(good, ipsi) vs (bad, ipsi)	–1.20(1.01)	–1.78(0.94)	4.52	<.0001	[0.33, 0.83]	0.82

Table S6. Summary of statistical test to compare the normalized neuronal activity of GPe neurons of cluster1 at saccade onset among conditions during choice task in Figure 3.

Cluster1						
parametric bootstrap test (n = 10,000)	*p*					
full model vs. null model	< .001					
post hoc (pairwise t–test, Bonferroni correction)	Mean (SD)	Mean (SD)	*t*	*p*	95% CI	effect size
**Scene1**						
(good, contra) vs (bad, contra)	1.72(0.58)	0.85(1.19)	5.88	<.0001	[0.58, 1.16]	1.09
(good, contra) vs (good, ipsi)	1.72(0.58)	0.81(0.88)	6.15	<.0001	[0.62, 1.20]	1.14
(good, contra) vs (bad, ipsi)	1.72(0.58)	0.35(1.35)	9.22	<.0001	[1.08, 1.66]	1.71
(bad, contra) vs (good, ipsi)	0.85(1.19)	0.81(0.88)	0.27	= 7.86 x 10^–1	[–0.25, 0.33]	0.05
(bad, contra) vs (bad, ipsi)	0.85(1.19)	0.35(1.35)	3.34	= 9.00 x 10^–4	[0.21, 0.79]	0.62
(good, ipsi) vs (bad, ipsi)	0.81(0.88)	0.35(1.35)	3.07	= 2.20 x 10^–3	[0.16, 0.75]	0.57
**Scene2**						
(good, contra) vs (bad, contra)	1.77(0.55)	0.82(1.17)	6.38	<.0001	[0.66, 1.24]	1.18
(good, contra) vs (good, ipsi)	1.77(0.55)	1.16(0.90)	4.09	<.0001	[0.32, 0.90]	0.76
(good, contra) vs (bad, ipsi)	1.77(0.55)	0.46(1.08)	8.78	<.0001	[1.01, 1.59]	1.63
(bad, contra) vs (good, ipsi)	0.82(1.17)	1.16(0.90)	–2.29	= 2.20 x 10^–2	[–0.63, –0.05]	–0.43
(bad, contra) vs (bad, ipsi)	0.82(1.17)	0.46(1.08)	2.40	= 1.66 x 10^–2	[0.06, 0.65]	0.45
(good, ipsi) vs (bad, ipsi)	1.16(0.90)	0.46(1.08)	4.69	<.0001	[0.41, 0.99]	0.87
**Scene3**						
(good, contra) vs (bad, contra)	1.74(0.56)	1.14(1.17)	4.07	= 1.00 x 10^–4	[0.31, 0.90]	0.76
(good, contra) vs (good, ipsi)	1.74(0.56)	0.96(1.00)	5.25	<.0001	[0.49, 1.07]	0.97
(good, contra) vs (bad, ipsi)	1.74(0.56)	0.54(1.23)	8.08	<.0001	[0.91, 1.49]	1.50
(bad, contra) vs (good, ipsi)	1.14(1.17)	0.96(1.00)	1.18	= 2.39 x 10^–1	[–0.12, 0.47]	0.22
(bad, contra) vs (bad, ipsi)	1.14(1.17)	0.54(1.23)	4.01	= 1.00 x 10^–4	[0.30, 0.89]	0.74
(good, ipsi) vs (bad, ipsi)	0.96(1.00)	0.54(1.23)	2.83	= 4.70 x 10^–3	[0.13, 0.71]	0.53
**Scene4**						
(good, contra) vs (bad, contra)	1.69(0.63)	1.13(1.07)	3.80	= 2.00 x 10^–4	[0.27, 0.86]	0.71
(good, contra) vs (good, ipsi)	1.69(0.63)	0.95(1.01)	5.05	<.0001	[0.46, 1.04]	0.94
(good, contra) vs (bad, ipsi)	1.69(0.63)	0.44(1.17)	8.48	<.0001	[0.97, 1.55]	1.57
(bad, contra) vs (good, ipsi)	1.13(1.07)	0.95(1.01)	1.25	= 2.12 x 10^–1	[–0.11, 0.48]	0.23
(bad, contra) vs (bad, ipsi)	1.13(1.07)	0.44(1.17)	4.68	<.0001	[0.40, 0.99]	0.87
(good, ipsi) vs (bad, ipsi)	0.95(1.01)	0.44(1.17)	3.43	= 6.00 x 10^–4	[0.22, 0.80]	0.64

Table S7. Summary of statistical test to compare the normalized neuronal activity of GPe neurons of cluster2 at saccade onset among conditions during choice task in Figure 3.

Cluster2						
parametric bootstrap test (n = 10,000)	*p*					
full model vs. null model	< .001					
post hoc<br>(pairwise t–test, Bonferroni correction)	Mean (SD)	Mean (SD)	*t*	*p*	95% CI	effect size
**Scene1**						
(good, contra) vs (bad, contra)	0.98(0.73)	–1.21(1.06)	14.36	<.0001	[1.89, 2.48]	2.19
(good, contra) vs (good, ipsi)	0.98(0.73)	0.15(0.99)	5.42	<.0001	[0.53, 1.12]	0.83
(good, contra) vs (bad, ipsi)	0.98(0.73)	–1.18(1.23)	14.22	<.0001	[1.86, 2.46]	2.17
(bad, contra) vs (good, ipsi)	–1.21(1.06)	0.15(0.99)	–8.94	<.0001	[–1.66, –1.06]	–1.36
(bad, contra) vs (bad, ipsi)	–1.21(1.06)	–1.18(1.23)	–0.14	= 8.86 x 10^–1	[–0.32, 0.28]	–0.02
(good, ipsi) vs (bad, ipsi)	0.15(0.99)	–1.18(1.23)	8.80	<.0001	[1.04, 1.64]	1.34
**Scene2**						
(good, contra) vs (bad, contra)	1.02(0.82)	–1.18(1.00)	14.48	<.0001	[1.90, 2.50]	2.21
(good, contra) vs (good, ipsi)	1.02(0.82)	0.33(1.10)	4.60	<.0001	[0.40, 1.00]	0.70
(good, contra) vs (bad, ipsi)	1.02(0.82)	–1.17(1.06)	14.45	<.0001	[1.90, 2.49]	2.20
(bad, contra) vs (good, ipsi)	–1.18(1.00)	0.33(1.10)	–9.88	<.0001	[–1.80, –1.20]	–1.50
(bad, contra) vs (bad, ipsi)	–1.18(1.00)	–1.17(1.06)	–0.03	= 9.73 x 10^–1	[–0.30, 0.29]	–0.01
(good, ipsi) vs (bad, ipsi)	0.33(1.10)	–1.17(1.06)	9.84	<.0001	[1.20, 1.80]	1.50
**Scene3**						
(good, contra) vs (bad, contra)	1.09(0.79)	–0.90(1.14)	13.13	<.0001	[1.70, 2.29]	2.00
(good, contra) vs (good, ipsi)	1.09(0.79)	0.38(1.08)	4.67	<.0001	[0.41, 1.01]	0.71
(good, contra) vs (bad, ipsi)	1.09(0.79)	–1.00(1.16)	13.75	<.0001	[1.79, 2.39]	2.10
(bad, contra) vs (good, ipsi)	–0.90(1.14)	0.38(1.08)	–8.45	<.0001	[–1.58, –0.99]	–1.29
(bad, contra) vs (bad, ipsi)	–0.90(1.14)	–1.00(1.16)	0.63	= 5.31 x 10^–1	[–0.20, 0.39]	0.10
(good, ipsi) vs (bad, ipsi)	0.38(1.08)	–1.00(1.16)	9.08	<.0001	[1.08, 1.68]	1.38
**Scene4**						
(good, contra) vs (bad, contra)	0.87(0.86)	–1.11(1.54)	13.03	<.0001	[1.68, 2.28]	1.99
(good, contra) vs (good, ipsi)	0.87(0.86)	0.23(1.04)	4.21	<.0001	[0.34, 0.94]	0.64
(good, contra) vs (bad, ipsi)	0.87(0.86)	–1.22(1.27)	13.76	<.0001	[1.79, 2.39]	2.10
(bad, contra) vs (good, ipsi)	–1.11(1.54)	0.23(1.04)	–8.82	<.0001	[–1.64, –1.04]	–1.34
(bad, contra) vs (bad, ipsi)	–1.11(1.54)	–1.22(1.27)	0.73	= 4.66 x 10^–1	[–0.19, 0.41]	0.11
(good, ipsi) vs (bad, ipsi)	0.23(1.04)	–1.22(1.27)	9.55	<.0001	[1.15, 1.75]	1.46

Table S8. Summary of statistical test to compare the normalized neuronal activity of GPe neurons of cluster3 at saccade onset among conditions during choice task in Figure 3.

Cluster3						
parametric bootstrap test (n = 10,000)	*p*					
full model vs. null model	< .001					
post hoc (pairwise t–test, Bonferroni correction)	Mean (SD)	Mean (SD)	*t*	*p*	95% CI	effect size
**Scene1**						
(good, contra) vs (bad, contra)	–0.90(1.00)	–1.75(0.85)	7.12	<.0001	[0.61, 1.08]	1.29
(good, contra) vs (good, ipsi)	–0.90(1.00)	–0.98(1.01)	0.66	= 5.11 x 10^–1	[–0.16, 0.31]	0.12
(good, contra) vs (bad, ipsi)	–0.90(1.00)	–1.82(0.91)	7.73	<.0001	[0.69, 1.15]	1.40
(bad, contra) vs (good, ipsi)	–1.75(0.85)	–0.98(1.01)	–6.46	<.0001	[–1.00, –0.54]	–1.17
(bad, contra) vs (bad, ipsi)	–1.75(0.85)	–1.82(0.91)	0.61	= 5.43 x 10^–1	[–0.16, 0.31]	0.11
(good, ipsi) vs (bad, ipsi)	–0.98(1.01)	–1.82(0.91)	7.07	<.0001	[0.61, 1.08]	1.28
**Scene2**						
(good, contra) vs (bad, contra)	–1.11(1.02)	–1.81(0.85)	5.87	<.0001	[0.47, 0.93]	1.06
(good, contra) vs (good, ipsi)	–1.11(1.02)	–1.16(1.05)	0.41	= 6.85 x 10^–1	[–0.19, 0.28]	0.07
(good, contra) vs (bad, ipsi)	–1.11(1.02)	–1.92(0.87)	6.76	<.0001	[0.57, 1.04]	1.23
(bad, contra) vs (good, ipsi)	–1.81(0.85)	–1.16(1.05)	–5.46	<.0001	[–0.88, –0.42]	–0.99
(bad, contra) vs (bad, ipsi)	–1.81(0.85)	–1.92(0.87)	0.92	= 3.59 x 10^–1	[–0.13, 0.35]	0.17
(good, ipsi) vs (bad, ipsi)	–1.16(1.05)	–1.92(0.87)	6.36	<.0001	[0.53, 1.00]	1.16
**Scene3**						
(good, contra) vs (bad, contra)	–1.06(1.00)	–1.78(0.90)	6.04	<.0001	[0.49, 0.95]	1.09
(good, contra) vs (good, ipsi)	–1.06(1.00)	–1.18(1.10)	1.02	= 3.10 x 10^–1	[–0.11, 0.36]	0.18
(good, contra) vs (bad, ipsi)	–1.06(1.00)	–1.69(0.92)	5.31	<.0001	[0.40, 0.87]	0.96
(bad, contra) vs (good, ipsi)	–1.78(0.90)	–1.18(1.10)	–5.03	<.0001	[–0.83, –0.37]	–0.91
(bad, contra) vs (bad, ipsi)	–1.78(0.90)	–1.69(0.92)	–0.73	= 4.65 x 10^–1	[–0.32, 0.15]	–0.13
(good, ipsi) vs (bad, ipsi)	–1.18(1.10)	–1.69(0.92)	4.30	<.0001	[0.28, 0.75]	0.78
**Scene4**						
(good, contra) vs (bad, contra)	–1.00(0.97)	–1.80(0.95)	6.76	<.0001	[0.57, 1.04]	1.22
(good, contra) vs (good, ipsi)	–1.00(0.97)	–1.15(1.04)	1.30	= 1.94 x 10^–1	[–0.08, 0.39]	0.24
(good, contra) vs (bad, ipsi)	–1.00(0.97)	–1.77(0.97)	6.44	<.0001	[0.53, 1.00]	1.17
(bad, contra) vs (good, ipsi)	–1.80(0.95)	–1.15(1.04)	–5.46	<.0001	[–0.88, –0.42]	–0.99
(bad, contra) vs (bad, ipsi)	–1.80(0.95)	–1.77(0.97)	–0.33	= 7.45 x 10^–1	[–0.27, 0.20]	–0.06
(good, ipsi) vs (bad, ipsi)	–1.15(1.04)	–1.77(0.97)	5.14	<.0001	[0.38, 0.85]	0.93

Table S9. Summary of statistical test to compare the normalized neuronal activity of GPe neurons of clusters 1, 2, and 3 among Return, Stay during choice task, and fixation task in Figure 4.

Cluster1						
parametric bootstrap test (n = 10,000)	*p*					
full model vs. null model	< .001					
post hoc (pairwise t–test, Bonferroni correction)	Mean (SD)	Mean (SD)	*t*	*p*	95% CI	effect size
Contra						
(Return, choice) vs (Stay, choice)	1.01(1.18)	0.73(1.00)	1.55	= 1.22 x 10^–1	[–0.08, 0.66]	0.30
(Return, choice) vs (Good, fixation)	1.01(1.18)	1.18(1.13)	–1.21	= 2.27 x 10^–1	[–0.73, 0.17]	–0.29
(Return, choice) vs (Bad, fixation)	1.01(1.18)	1.09(1.13)	–0.85	= 3.99 x 10^–1	[–0.65, 0.26]	–0.20
(Stay, choice) vs (Good, fixation)	0.73(1.00)	1.18(1.13)	–2.40	= 1.72 x 10^–2	[–1.04, –0.10]	–0.59
(Stay, choice) vs (Bad, fixation)	0.73(1.00)	1.09(1.13)	–2.05	= 4.18 x 10^–2	[–0.95, 0.02]	–0.51
(Good, fixation) vs (Bad, fixation)	1.18(1.13)	1.09(1.13)	0.33	= 7.44 x 10^–1	[–0.59, 0.42]	0.09
Ipsi						
(Return, choice) vs (Stay, choice)	0.41(1.35)	0.34(1.16)	0.34	= 7.38 x 10^–1	[–0.32, 0.45]	0.07
(Return, choice) vs (Good, fixation)	0.41(1.35)	–0.12(1.41)	1.87	= 6.31 x 10^–2	[–0.02, 0.88]	0.45
(Return, choice) vs (Bad, fixation)	0.41(1.35)	–0.22(1.30)	2.30	= 2.21 x 10^–2	[0.08, 0.98]	0.55
(Stay, choice) vs (Good, fixation)	0.34(1.16)	–0.12(1.41)	1.48	= 1.39 x 10^–1	[–0.12, 0.84]	0.38
(Stay, choice) vs (Bad, fixation)	0.34(1.16)	–0.22(1.30)	1.89	= 5.95 x 10^–2	[–0.02, 0.94]	0.48
(Good, fixation) vs (Bad, fixation)	–0.12(1.41)	–0.22(1.30)	0.39	= 6.98 x 10^–1	[–0.41, 0.60]	0.10
Cluster2						
parametric bootstrap test (n = 10,000)	*p*					
full model vs. null model	< .001					
post hoc (pairwise t–test, Bonferroni correction)	Mean (SD)	Mean (SD)	*t*	*p*	95% CI	effect size
Contra						
(Return, choice) vs (Stay, choice)	–1.11(1.08)	–0.92(1.11)	–1.13	= 2.59 x 10^–1	[–0.45, 0.12]	–0.18
(Return, choice) vs (Good, fixation)	–1.11(1.08)	0.35(1.45)	–7.73	<.0001	[–1.75, –1.04]	–1.56
(Return, choice) vs (Bad, fixation)	–1.11(1.08)	0.13(1.12)	–6.50	<.0001	[–1.53, –0.82]	–1.31
(Stay, choice) vs (Good, fixation)	–0.92(1.11)	0.35(1.45)	–6.60	<.0001	[–1.60, –0.87]	–1.37
(Stay, choice) vs (Bad, fixation)	–0.92(1.11)	0.13(1.12)	–5.41	<.0001	[–1.38, –0.64]	–1.13
(Good, fixation) vs (Bad, fixation)	0.35(1.45)	0.13(1.12)	1.12	= 2.65 x 10^–1	[–0.17, 0.61]	0.25
Ipsi						
(Return, choice) vs (Stay, choice)	–1.13(1.22)	–0.99(1.12)	–0.89	= 3.72 x 10^–1	[–0.42, 0.16]	0.14

(Return, choice) vs (Good, fixation)	–1.13(1.22)	–0.14(1.26)	–5.12	<.0001	[–1.28, –0.57]	–1.03
(Return, choice) vs (Bad, fixation)	–1.13(1.22)	–0.08(0.96)	–5.48	<.0001	[–1.35, –0.63]	–1.10
(Stay, choice) vs (Good, fixation)	–0.99(1.12)	–0.14(1.26)	–4.21	<.0001	[–1.17, –0.42]	–0.89
(Stay, choice) vs (Bad, fixation)	–0.99(1.12)	–0.08(0.96)	–4.55	<.0001	[–1.23, –0.49]	–0.96
(Good, fixation) vs (Bad, fixation)	–0.14(1.26)	–0.08(0.96)	0.32	= 7.46 x 10^–1	[–0.45, 0.33]	–0.07
Cluster3						
parametric bootstrap test (n = 10,000)	*p*					
full model vs. null model	< .001					
post hoc (pairwise t–test, Bonferroni correction)	Mean (SD)	Mean (SD)	*t*	*p*	95% CI	effect size
Contra						
(Return, choice) vs (Stay, choice)	–1.71(0.86)	–1.67(0.84)	–0.34	= 7.31 x 10^–1	[–0.36, 0.25]	–0.07
(Return, choice) vs (Good, fixation)	–1.71(0.86)	–0.15(1.11)	–8.62	<.0001	[–1.83, –1.15]	–1.85
(Return, choice) vs (Bad, fixation)	–1.71(0.86)	–0.24(1.08)	–8.10	<.0001	[–1.74, –1.06]	–1.74
(Stay, choice) vs (Good, fixation)	–1.67(0.84)	–0.15(1.11)	–7.93	<.0001	[–1.79, –1.08]	–1.79
(Stay, choice) vs (Bad, fixation)	–1.67(0.84)	–0.24(1.08)	–7.43	<.0001	[–1.70, –0.99]	–1.67
(Good, fixation) vs (Bad, fixation)	–0.15(1.11)	–0.24(1.08)	0.48	= 6.34 x 10^–1	[–0.28, 0.46]	0.11
Ipsi						
(Return, choice) vs (Stay, choice)	–1.73(0.83)	–1.57(0.86)	–1.39	= 1.65 x 10^–1	[–0.10, 0.56]	–0.29
(Return, choice) vs (Good, fixation)	–1.73(0.83)	–0.25(0.93)	–8.13	<.0001	[–1.75, –1.07]	–1.75
(Return, choice) vs (Bad, fixation)	–1.73(0.83)	–0.19(0.90)	–8.48	<.0001	[–1.81, –1.13]	–1.82
(Stay, choice) vs (Good, fixation)	–1.57(0.86)	–0.25(0.93)	–6.11	<.0001	[–1.55, –0.80]	–1.46
(Stay, choice) vs (Bad, fixation)	–1.57(0.86)	–0.19(0.90)	–6.42	<.0001	[–1.61, –0.86]	–1.54
(Good, fixation) vs (Bad, fixation)	–0.25(0.93)	–0.19(0.90)	0.32	= 7.52 x 10^–1	[–0.43, 0.31]	0.07

Table S10. Summary of statistical test to compare the effects of CPP + NBQX injection into GPe during choice task in Figure 5.

Injection during choice task				
parametric bootstrap test (n = 10,000)	*p*			
full model vs. null model	< .0001			
post hoc (pairwise t–test, Bonferroni correction)	Mean (ms) (SD)	Mean (ms) (SD)	*z*	*p*
CPP+NBQX Contra Good pre vs. post	193.53 (10.26)	235.13 (15.33)	7.77	<.0001
Saline Contra Good pre vs. post	190.80 (9.16)	188.67 (9.89)	–0.42	= 6.71 x 10^–1
CPP+NBQX Contra Bad pre vs. post	281.07 (34.01)	301.87 (51.00)	3.34	= 9.00 x 10^–4
Saline Contra Bad pre vs. post	277.33 (24.25)	271.60 (22.58)	–0.95	= 3.43 x 10^–1
CPP+NBQX Ipsi Good pre vs. post	195.93 (12.03)	190.60 (11.55)	–1.05	= 2.93 x 10^–1
Saline Ipsi Good pre vs. post	196.27 (8.53)	191.87 (10.93)	–0.87	= 3.87 x 10^–1
CPP+NBQX Ipsi Bad pre vs. post	281.00 (33.55)	266.87 (42.03)	–2.34	= 1.94 x 10^–2
Saline Ipsi Bad pre vs. post	279.60 (32.37)	273.33 (29.78)	–1.03	= 3.02 x 10^–1

Table S11. Summary of statistical test to compare the effects of CPP + NBQX injection into GPe while monkeys chose actions for Bad object during Choice task in Figure 5.

Injection into SNr during Choice task				
Accept Bad object				
parametric bootstrap test (n = 10,000)	*p*			
full model vs. null model	= 1.00			
Return for Bad object				
parametric bootstrap test (n = 10,000)	*p*			
full model vs. null model	< .001			
post hoc<br>(pairwise t–test, Bonferroni correction)	Mean (%)<br>(SD)	Mean (%)<br>(SD)	*z*	*p*
CPP+NBQX<br>Return Contra Bad pre vs. post	75.89<br>(14.46)	43.22<br>(35.89)	–<br>12.62	<.0001
Saline<br>Return Contra Bad pre vs. post	80.54<br>(13.98)	83.76<br>(7.93)	1.65	= 9.87 x<br>10^–2
CPP+NBQX<br>Return Ipsi Bad pre vs. post	74.06(<br>11.99)	64.75<br>(21.98)	–3.79	= 2.00 x<br>10^–4
Saline<br>Return Ipsi Bad pre vs. post	77.26<br>(14.22)	81.63<br>(10.62)	2.09	= 3.66 x<br>10^–2
Stay for Bad object				
parametric bootstrap test (n = 10,000)	*p*			
full model vs. null model	< .001			
post hoc<br>(pairwise t–test, Bonferroni correction)	Mean (%)<br>(SD)	Mean (%)<br>(S.D.)	*z*	*p*
CPP+NBQX<br>Return Contra Bad pre vs. post	23.94<br>(14.21)	56.62<br>(35.72)	12.68	<.0001
Saline<br>Return Contra Bad pre vs. post	19.46<br>(13.98)	15.80<br>(8.04)	–1.86	= 6.25 x<br>10^–2
CPP+NBQX<br>Return Ipsi Bad pre vs. post	25.58<br>(12.22)	29.50<br>(24.52)	1.54	= 1.23 x<br>10^–1
Saline<br>Return Ipsi Bad pre vs. post	22.74<br>(14.22)	18.37<br>(10.62)	–2.13	= 3.28 x<br>10^–2
